# Technical tips for EUS for pancreatic lesions in patients undergoing total gastrectomy and Roux-en-Y reconstruction (with videos)

**DOI:** 10.1097/eus.0000000000000104

**Published:** 2025-02-21

**Authors:** Tatsuya Koshitani, Kumpei Kadosaka, Hiroshi Takihara, Takahiro Takemoto

**Affiliations:** 1 Department of Gastroenterology, Uji Tokushukai Medical Center, Kyoto, Japan; 2 Department of Gastroenterology, Uji Tokushukai Medical Center, Kadosaka Clinic, Kyoto, Japan.

EUS for pancreatic lesions in patients with altered gastrointestinal anatomy has technical challenges.^[1–3]^ This study aims to provide technical tips for EUS for pancreatic lesions in patients who underwent total gastrectomy and Roux-en-Y reconstruction. EUS and fluoroscopic imaging were presented during pancreatic observation in such patients, and EUS-guided fine-needle aspiration (EUS-FNA) was performed. A convex echoendoscope (GF-UCT-260; Olympus) and EUS video processors (EU-ME2 and ME3; Olympus) were used.

First, the echoendoscope was positioned proximal to the efferent jejunal loop. Pancreatic visualization from the body to the tail was achieved using a clockwise rotation, similar to normal anatomical conditions, whereas an anticlockwise rotation was used for visualization from the tail to the body. Next, the echoendoscope was advanced slightly deeper into the efferent loop to observe the pancreatic head. Visualization of the pancreatic head required adjusting the position of echoendoscope while rotating the endoscope. Pancreatic observation from the head to the tail was then feasible using a clockwise rotation while gradually withdrawing the echoendoscope. Fluoroscopic imaging confirmed that the echoendoscope was positioned at a deeper site in the efferent loop, and the tip of the scope was angulated [Figure 1 and Video 1].

**Supplementary Videos**

Video 1: EUS and fluoroscopic imaging during pancreatic observation after total gastrectomy and Roux-en-Y reconstruction;

**Figure 1:**
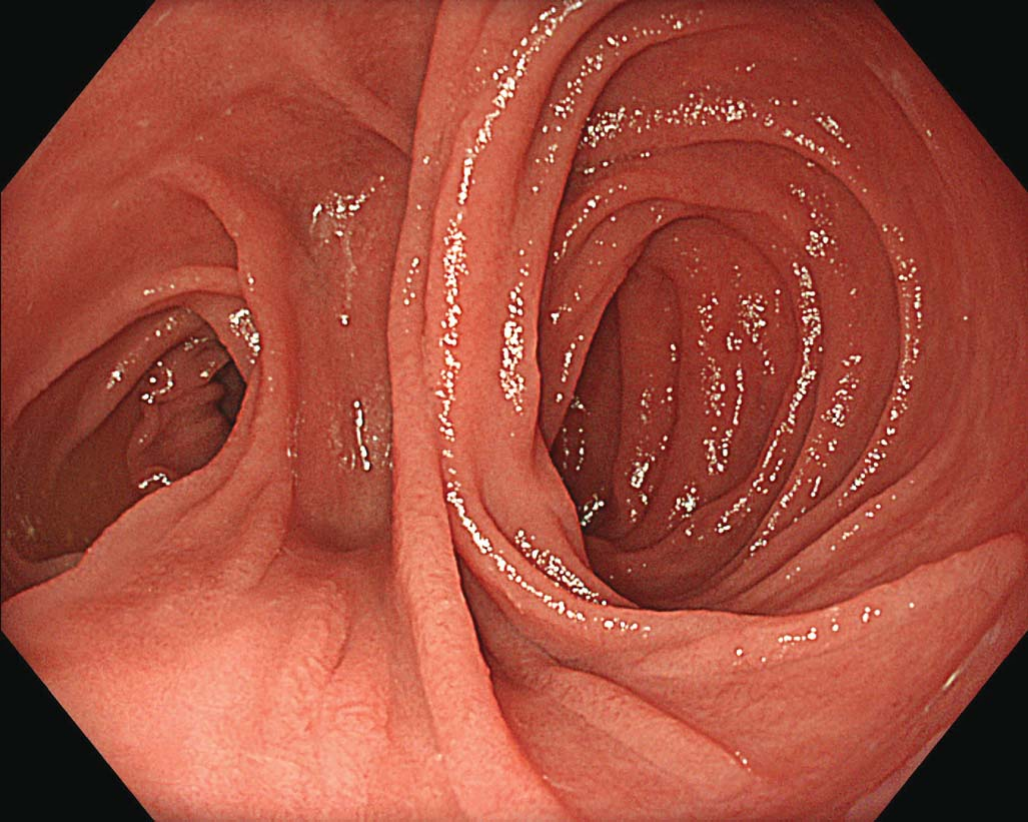
Esophagojejunal anastomosis in a patient undergoing total gastrectomy and Roux-en-Y reconstruction. Videos are only available at the official website of the journal (www.eusjournal.com).

Herein, we present 3 cases: cases 1 and 2 involved heterogeneous hypoechoic lesions with irregular margins in the pancreatic tail and head, respectively. EUS-FNA was performed, and pathological examination revealed adenocarcinoma in each case. In case 3, EUS was performed to investigate a dilated common bile duct. Incidentally, a multilocular cystic lesion was found in the pancreatic head and was diagnosed as a branch-duct intraductal papillary mucinous neoplasm [Figures 2, 3, 4, and Video 2].

**Figure 2:**
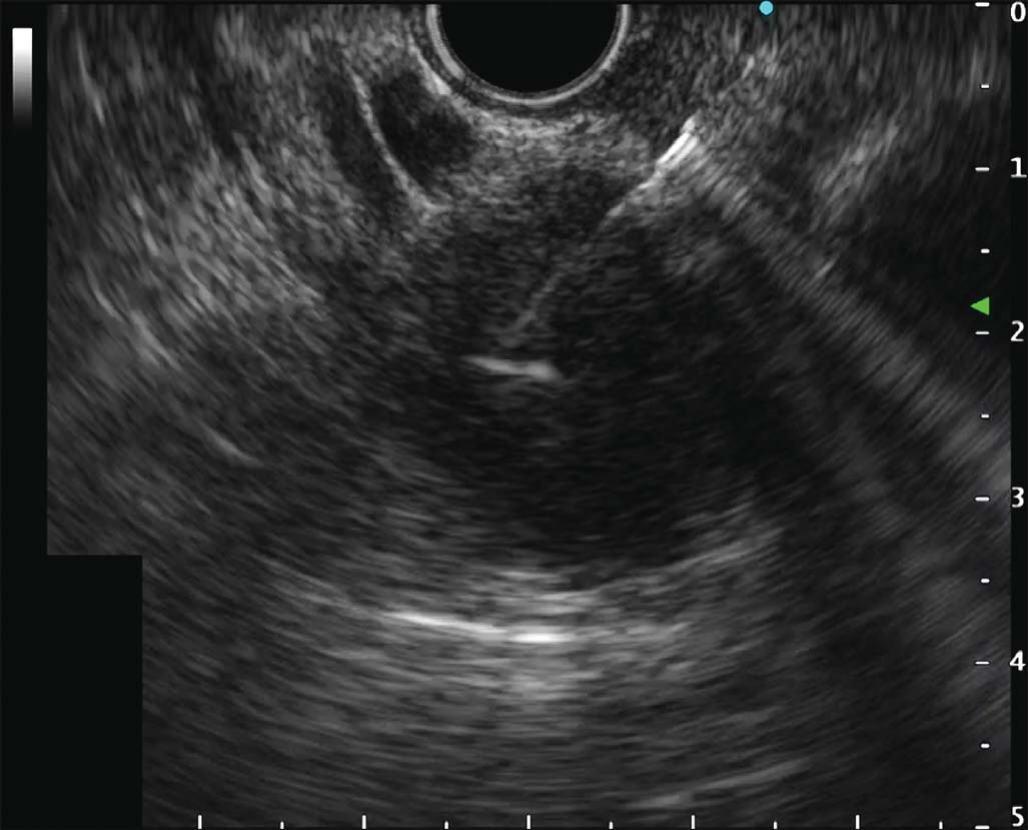
Case 1: Pancreatic adenocarcinoma in the pancreatic tail. Endoscopic ultrasound–guided fine-needle aspiration (EUS-FNA) was performed.

**Figure 3:**
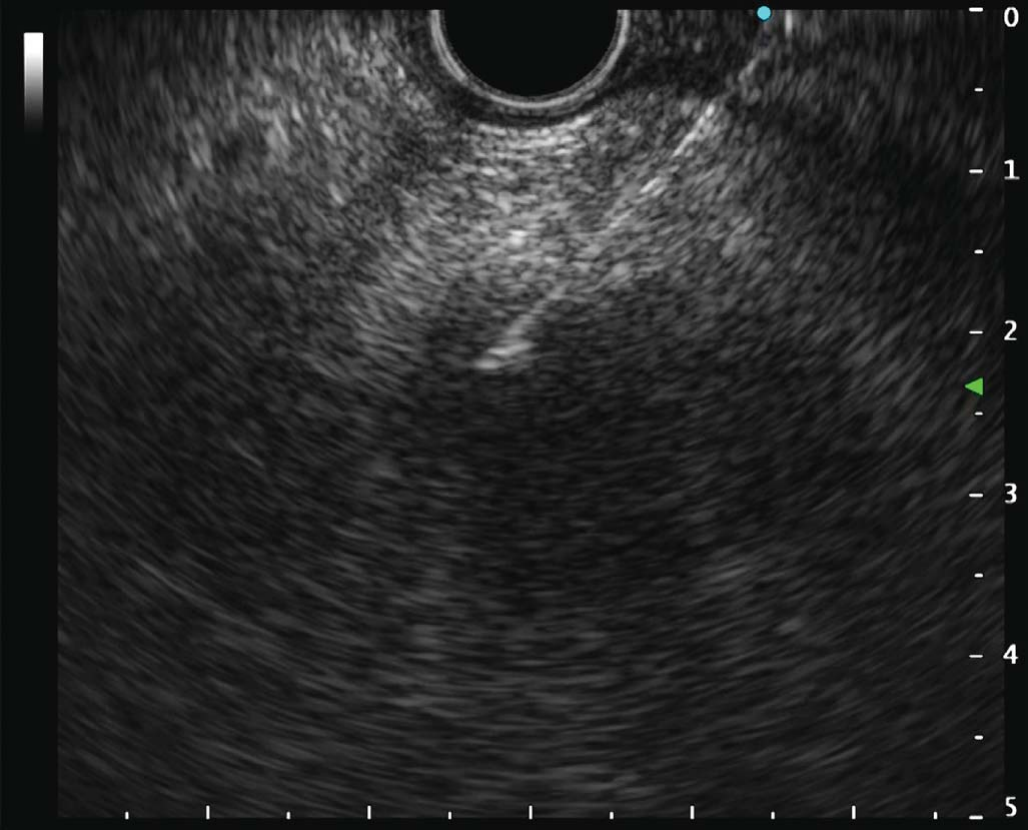
Case 2: Pancreatic adenocarcinoma in the pancreatic head. EUS-FNA was performed.

**Figure 4:**
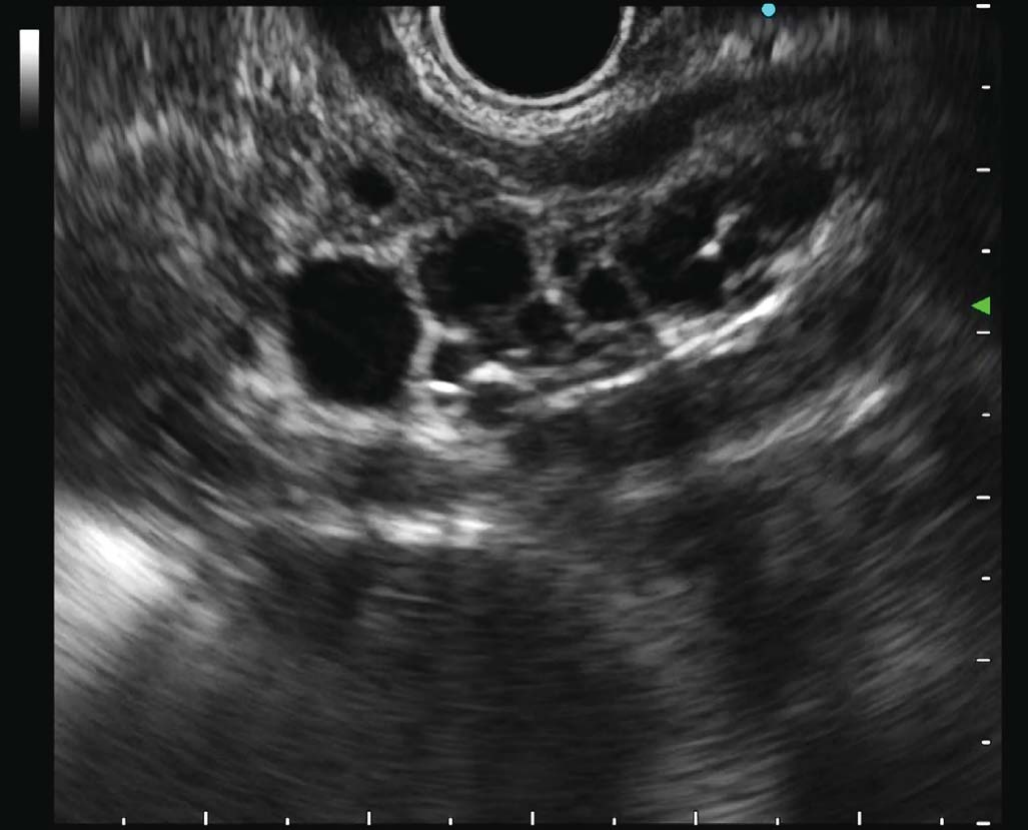
Case 3: Branch-duct intraductal papillary mucinous neoplasm in the pancreatic head.

EUS for pancreatic lesions in patients who had underwent total gastrectomy and Roux-en-Y reconstruction remains challenging, particularly for pancreatic head lesions. However, such examination could be facilitated by initiating EUS at a slightly deeper site in the efferent loop. This technique appears applicable to most patients with Roux-en-Y reconstruction. Nevertheless, careful echoendoscope handling is essential to minimize the risk of injury to the efferent loop. Once a pancreatic lesion is identified, conventional techniques for EUS-FNA can be applied.

## Ethical Approval

The study was conducted according to the guidelines of the Declaration of Helsinki.

## Informed Consent

Informed consent was obtained from all the patients for the study.

## Conflicts of Interest

The authors indicated no potential conflicts of interest.

## Author Contributions

T.K. designed and directed the study and wrote the manuscript. K.K. assisted with the procedure. All authors critically revised the manuscript and approved the final manuscript.

## References

[1] Wilson JA Hoffman B Hawes RH, . EUS in patients with surgically altered upper GI anatomy. *Gastrointest Endosc* 2010;72:947–953. 21034896 10.1016/j.gie.2010.07.016

[2] Brozzi L Petrone MC Poley JW, . Outcomes of biliopancreatic EUS in patients with surgically altered upper gastrointestinal anatomy: A multicenter study. *Endosc Int Open* 2020;8:E869–E876. 32617391 10.1055/a-1161-8713 PMC7297615

[3] Tanaka K Hayashi T Utsunomiya R, . Endoscopic ultrasound-guided fine needle aspiration for diagnosing pancreatic mass in patients with surgically altered upper gastrointestinal anatomy. *Dig Endosc* 2020;32:967–973. 31912558 10.1111/den.13625

